# From Morphology to Neural Information: The Electric Sense of the Skate

**DOI:** 10.1371/journal.pcbi.0030113

**Published:** 2007-06-15

**Authors:** Marcelo Camperi, Timothy C Tricas, Brandon R Brown

**Affiliations:** 1 Department of Physics, University of San Francisco, San Francisco, California, United States of America; 2 Department of Zoology, University of Hawaii at Manoa, Honolulu, Hawaii, United States of America; 3 Hawaii Institute of Marine Biology, University of Hawaii at Manoa, Honolulu, Hawaii, United States of America; University College London, United Kingdom

## Abstract

Morphology typically enhances the fidelity of sensory systems. Sharks, skates, and rays have a well-developed electrosense that presents strikingly unique morphologies. Here, we model the dynamics of the peripheral electrosensory system of the skate, a dorsally flattened batoid, moving near an electric dipole source (e.g., a prey organism). We compute the coincident electric signals that develop across an array of the skate's electrosensors, using electrodynamics married to precise morphological measurements of sensor location, infrastructure, and vector projection. Our results demonstrate that skate morphology enhances electrosensory information. Not only could the skate locate prey using a simple population vector algorithm, but its morphology also specifically leads to quick shifts in firing rates that are well-suited to the demonstrated bandwidth of the electrosensory system. Finally, we propose electrophysiology trials to test the modeling scheme.

## Author Summary

The electric sense appears in a variety of animals, from the shark to the platypus, and it facilitates short-range prey detection where environments limit sight. Typically, hundreds or thousands of sensors work in concert. In skates, rays, and sharks, each electrosensor includes a small, innervated bulb, with a thin, gel-filled canal leading to a surface pore. While experiments have mapped single electrosensor activity, the mechanisms that integrate neural input from multiple electrosensors are still largely unknown. Here, we model the response of a precisely mapped subset of electrosensors responding in concert for a skate moving near stationary prey. Just as two ears help locate sound via time and intensity differences, we ask how a bilateral electrosensor array can contribute to electrical scene analysis. Our results show that the sensor array provides rich data for precise prey location, tuned by the morphology to render certain events, like the point of closest approach, “loud and clear.” This proof of principle makes a significant step in understanding the electric sense processing, and we recommend future experiments to compare and assess functions for the diversity of arrays found in other sharks and rays.

## Introduction

Sensor placement and arrangement are crucial for any sensory system used to locate the distance and bearing of a stimulus source, and this is true for a variety of modalities. In addition to stereo vision in much of the animal kingdom, echolocation (e.g., the barn owl) and mechanosensory location (e.g., the sand scorpion) uses multiple sensors to locate prey with requisite precision [1–3].

Electrosensitive vertebrates, including monotremes (e.g., the platypus), siluriforms (e.g., the catfish), osteoglossomorphs (e.g., the knifefish), and chondrostreans (e.g., the sturgeon and the paddlefish), typically possess large populations of independent electrosensitive organs. Obviously, multiple organs enhance signal-to-noise ratio by an increased number of simultaneous measurements since environmental electrical signatures of biological relevance are often weak, even at close range. However, these animals may also use the sensor population to determine the location of electric sources. In the case of the black ghost knifefish, *Apteronotus albifrons,* the surface receptors simply report magnitudes of electric field, and the contrast of signal magnitudes across the body facilitate the location of nearby objects and the capture of prey [4,5].

**Figure 1:**
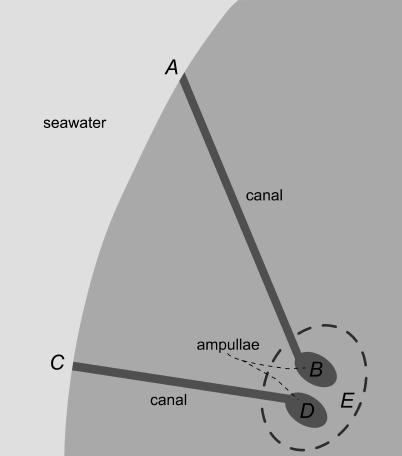
Simplified Schematic Depicting Two Ampullae within a Single Cluster, with Their Associated Canals and Pores Points A and C denote two pores, leading via gel-filled canals to their respective ampullae. Points B and D denote the inner ampullae, referencing electric potentials on the apical sides of the respective sensory epithelia. Point E is a common reference for the basal sides of ampullae within the cluster. The model used here emphasizes the potential differences arising along the internal gel of the narrow canals as driving the apical potentials, which lead to excitation or inhibition based on their relation to the relatively constant basal potential at point E (see text).

The elasmobranch fishes (sharks, skates, and rays) possess an electrosensory system used to detect prey, possibly navigate with respect to magnetic fields, and locate mates [6–9]. The system includes hundreds or thousands of separate electrosensor units known as the ampullae of Lorenzini. The ampullae are often tightly clustered, but each is linked to an individual pore on the surface of the body via a long, gel-filled canal (see Figure 1), and the pores are widely distributed (on the head and pectoral fins in skates and rays).

Each ampulla is able to code minute electrical fluctuations into discharge patterns of primary afferent nerves [10]. The sensing cells of an ampulla's epithelium detect electric potential differences between its apical side within the ampulla, and its basal side outside the ampulla. A sudden drop in apical-side potential with respect to the basal potential leads to an increased firing rate for the associated nerves, while an increase in that potential difference leads to an inhibited firing rate [11]. More recent measurements also delineate organ-specific and frequency-specific gain functions [12,13]. The amplification mechanism of the sensory epithelium is not completely understood, but persuasive models exist [11].

While previous studies have focused on the response properties of single electroreceptors, few have explored the simultaneous response of electroreceptor populations to biological stimuli. This aspect is especially relevant since the pores and canals associated with the electroreceptors display great geometrical variation within a given organism and among species [14]. In terms of higher processing, output from principal cells in the dorsal octavolateralis nucleus (DON) of the skate hindbrain showed systematic responses to oscillating dipole stimulation that were twice as strong as responses to uniform body-wide fields [15]. Hence, it is important to understand the coincident responses of the electrosensory periphery to electric stimuli to understand how central processing mechanisms integrate peripheral receptor responses and affect behavior.

Kalmijn has proposed an algorithm for elasmobranchs approaching stationary prey via the electric sense [16]. In this model, the hunting elasmobranch simply maintains its orientation with respect to the dipole field of the prey. Geometrically, this means that the elasmobranch will always arrive at the source, even if this sometimes means an inefficient, spiraling path. In an initial simulation effort, one of us showed that the Kalmijn algorithm would typically mean that an elasmobranch moves in such a way to reinforce the signal received by each electroreceptor [17]. However, recent behavioral analyses of sharks approaching artificial electric dipoles show that the animals usually exhibit sharp turns toward the electric field source [18]. Thus, it appears that information regarding the source's location and/or the decision to approach the source is developed rather quickly by the predator. These observations are not necessarily at odds with the Kalmijn algorithm, however. The algorithm could guide a creature for a brief period of time until an overwhelming strength of signal among the sensor population helps it establish the exact location of the source.

From the electrosensors, primary afferents project to principal cells of the DON in the medulla [19]. Ascending efferent neurons in the skate have a strong ascending projection from the medulla to the contralateral midbrain, where they converge in the lateral mesencephalic nucleus and the tectum, combining there with other sensory inputs [20]. Self-generated electrical signal subtraction has been convincingly documented in the elasmobranch DON [19,21]. While the afferent fibers show vigorous reactions to an elasmobranch's own ventilatory movements, the ascending efferent neurons demonstrate so-called common-mode suppression. In addition, electrical noise created by self-generated movements may be cancelled by an adaptive filter via anti-Hebbian learning of ascending efferent neurons in the hindbrain [22]. (By anti-Hebbian we mean responses to familiar, repetitive stimuli are suppressed in favor of novel stimuli.) However, how the combined neural responses from the periphery are processed by the DON, the midbrain, and higher centers to determine prey headings is unexplored in sharks and batoids.

Here, we model the system-wide signals and neural responses of an elasmobranch fish. In contrast to a prior modeling effort [17], we now use precise, biologically relevant morphological measurements and move beyond electrical signals within the ampullae to the population codes of neural information available to the elasmobranch central nervous system. The canals in sharks' electrosensory systems thoroughly map a 3-D space. While this is computationally straightforward, data representations and interpretations are cumbersome. Therefore, we examine several scenarios for the peripheral electrosense of the dorso–ventrally flattened barndoor skate, *Raja laevis,* moving near an electric dipole. Adults are typically found at significant benthic depths along mud floor troughs, where they skim along the dark sea floor to feed on large crustaceans, mollusks, worms, and flatfish [23]. We model a barndoor skate moving near an electric dipole to represent the approach to a stationary prey, but these computations can also represent a moving dipole source that approaches a stationary skate. On average, the barndoor skate possesses a greater number of electroreceptors than other skate species [24]. The peripheral electrosensory geometry is precisely mapped [14], and it presents an almost perfectly 2-D system. Where prior efforts stopped at voltage calculations [17], here we take two further steps: we assess afferent spike trains of the peripheral system, and present a basic model that averages these afferent inputs to ascertain the location of a bioelectric field's source.

## Materials and Methods

### Computation of Signals

**Figure 2:**
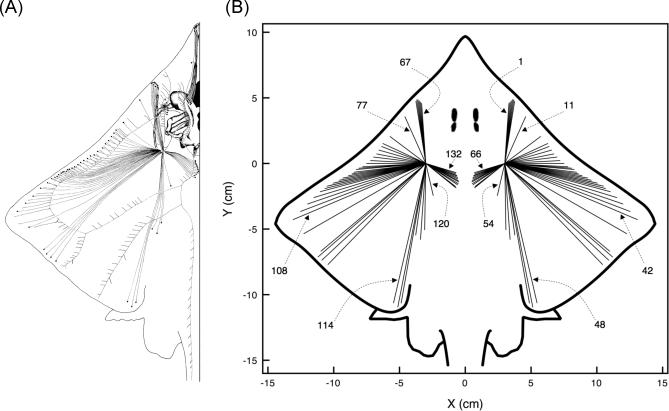
Canal Projections from the Dorsal Hyoid Ampullae of Raja laevis (A) Adapted from [14]. (B) As used in the present modeling work. Here, ampullary clusters are treated as a single point for simplicity. (B) also presents the canal numbering used in plots in this study. The 132 dorsal hyoid canals in the barndoor skate morphology are numbered consecutively, with canals 1 to 66 in the right cluster, and canals 67 to 132 in the left cluster. As seen in the figure, the first canal in each cluster is the one pointing in the most forward direction, 4° off the longitudinal axis. The other canals in each cluster are numbered consecutively, clockwise for the right cluster, and counterclockwise for the left cluster. Locations of pores and ampullae used in modeling match those in the actual fish (A). In terms of potential differences between an ampulla and a pore for a given canal (which is what our model emphasizes), the physics of electromagnetism tells us that the actual shape of the canals is immaterial. Thus, we simply represent them as straight lines.

For the case of the skate moving with respect to a live source, we first compute the realistic voltage signals that develop in the ampullae of Lorenzini using the skate's measured canal geometry. In this work, we concentrate on the bilateral canals of the hyoid clusters that project to the dorsal surface of the animal (see Figure 2). Of approximately 1,400 total ampullary canals in this species, we choose to focus on the dorsal hyoid cluster for two main reasons. First, we narrow the focus for the sake of clarity and lucid data management; we show results for the signals arising from just 132 ampullae. Second, we choose the dorsal hyoid clusters because they exhibit both the greatest canal lengths (corresponding to the lowest signal thresholds; [12]) and also the greatest variation of canal orientations when compared with other clusters. These clusters appear to be best suited for long-range detection when compared with the short canals of the rostral surfaces and the mandibular cluster on the jaw that presumably assists positioning for the final stages of feeding.

**Figure 3:**
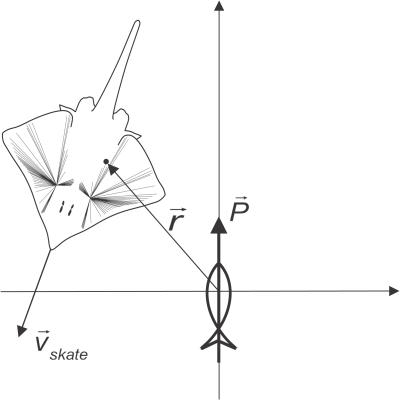
Skate Moving in a Source's Frame of Reference A point in the skate (a pore or an ampulla) is labeled by the vector *r* in the source's reference frame.

Though some authors have treated each canal as an equipotential or cable-like contact, linking the pore directly to its associated ampulla [25–27], we agree with the viewpoint that significant potential differences can and will develop within the length of the canals [28]. Here, consistent with an earlier simulation [17], we emphasize the potential difference that develops between an electroreceptor and its associated pore, due to the electric field originating in the physical electric dipole *P* of small bioelectric source (see Figure 3).

The origin for our computations sits at the source electric dipole. The potential at all other points, referenced by the position vector *r*, is computed for an ideal electric dipole [29],

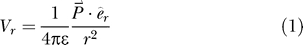



Here, *P* is the source's dipole moment vector, which includes the orientation direction of the source; *r* is the distance from the source to the point of interest; *e_r_* is the unit vector pointing from the source to the point of interest; and *ɛ* is the static permittivity of seawater (see Figure 3). Our entire computation is 2-D, approximating the dorso–ventrally flattened skate skimming the ocean floor in search of prey.

As originally noted by Murray, maximum excitatory or inhibitory response occurs when an electric field vector is parallel or antiparallel to a canal [10]. This geometrical observation is fundamental to our model, as this alignment maximizes the potential difference between a pore and its associated ampulla.

We refer to the geometry of Figure 1 to describe our assumptions and computations of relevant potential differences in the electrosensory periphery. There are five geometric points of interest, labeled A through E. The two ampulla and respective canals belong to the same cluster here. We assume that the basal voltage will be shared for each ampulla in a cluster (Figure 1, point E, within the sac that surrounds the cluster). Far from a dipole or other electric source, we assume all potentials at pores and ampullae to be more or less equal, and the two organs will exhibit the same resting firing rate. Fluctuations in the ampulla relative to the electric potential at point E will determine excited or inhibited firing rates for each organ.

Since we treat the potential at Figure 1, point E, *V_E_*, as a constant for all organs within a given cluster, the crucial quantities become the apical electric potentials *within* the ampullae (at points B and D in Figure 1). In what manner would these electric potentials vary? Will they communicate electric potentials from their pores (Figure 1, points A and C)? Recent electrical measurements of the gel demonstrate that the gel-filled canals do not provide good electrical contact between pores and ampullae [30]. The gel exhibits much stronger capacitive properties (charge storage) than seawater or generic collagen gels, suggesting a canal is better suited to sustaining electric potential *differences* than it is to bringing its two ends to quick electric equilibrium [30]. In fact, the relative electric resistance along either canal (e.g., from points A to B, or from points C to D; Figure 1) is more than 100 times greater than the electrical resistance between the pores A and C (in the semi-infinite seawater medium) [30]. Based on these measurements, and based on the observations of Murray regarding the crucial nature of geometric alignment between the canal and applied electric fields [10], we believe that the potential differences evolving along canals will provide crucial voltages in the system, with a driving influence for the apical surface of the sensory epithelium. The electric potentials between nearby pores would approach the same electric potential before the electric potentials at either ends of a canal would equilibrate.

Hence, as a skate draws close to an electric source, the sharply inhomogeneous source field will set up significant differences along the canals, depending on their length, angular orientation, and relative distance from the source [17]. Clustered ampullae will thereby be allowed to develop distinct firing responses, as each develops distinct apical potentials. By treating the basal electric potential (*V_E_*, using the geometry of Figure 1) as a constant for all ampullae in a given cluster, the model's chief simplification is a focus on the voltages that arise along the interior length of the canals. These lead to variations at the apical surface of the sensory epithelium, and with the basal potential treated as a constant, the apical variations drive the resulting firing responses.

Our model treats the sensory epithelium as a “black box” that translates transepithelial electrical fluctuations into firing rate alterations (see Analysis below). We do not imply that it plays a minor role or that its electrical characteristics are trivial. The epithelium's behavior is best described by multiple circuit elements acting in concert, with some functioning as a strong impediment to current flow; as a distinct electrical entity, its measured impedance exhibits great variation, presumably resulting from the behavior and condition of epithelial ion channels [11]. These details are not germane to our model, and we rely on the epithelium's measured and predictable response to electrical stimuli.

At successive moments in time, as the skate moves with respect to the source dipole in close range (within 1 m of distance), we assign an electrical signal *V_signal_* to each electrosensor by computing the electric potential at the various pores and associated ampullae, and then using the expression

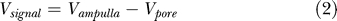
in which *V_pore_* is the potential at the pore and *V_ampulla_* is the potential in the internal ampulla chamber (apical side of the sensing cells). For instance, using Figure 1, the electric signals are computed as the relative potential differences respectively from points B to A and from points D to C. Here, both *V_ampulla_* and *V_pore_* are computed using the classical expression for the dipole electric potential. This is exactly equivalent to the path integral treatment used in a previous effort [17]. Given the simplifications described above, we do not pretend that our data will be the *precise* signals leading to firing alterations in the skate—however, we believe our computations capture the dominant component of firing alterations that arise when the skate moves with respect to an electric field source.

For the source dipole we use a magnitude that is consistent with bioelectric fields measured for small prey, with dipole values in the 10^−15^−10^−16^ Cm range [28]. While actual bioelectric fields do not conform exactly to an ideal dipole field, using such fields makes for an excellent approximation for distances greater than the physical extent of the source itself. When comparing the ideal dipole field magnitude to the slightly more realistic physical dipole (separating the positive and negative charge centers), we find that the ideal case gives values nearly identical to the physical dipole as long as the skate-to-source separation is more than two to three times the size of the source. For instance, if the source were a bivalve with 3 cm separating its relatively positive and negative charge centers, the ideal dipole approximation would be quite accurate as long as a skate was more than 6–9 cm away. This approximation will be valid for the cases explored here.

In early simulations, one of us used a dipole moment strength of 5.6 × 10^−16^ Cm [17]. This produced values for *V*_signal_ in the range of tens to hundreds of nanovolts for the skate–source distances of interest. These low values congregate at the very center of the empirical gain function for computing afferent firing rates (see Analysis). In this region, the empirical fitting is most speculative. Therefore, we increased the dipole moment strength for the current simulations, using a value of 3 × 10^−15^ Cm (i.e., around five times the original choice). This is not far off from measured values for small prey sources [28], and renders values for *V_signal_* in the low microvolt range, where the translation to firing rate alterations is well mapped.

### Analysis

We translate ampullary signals from Equation 2 into firing rate alterations of the primary afferent neurons associated with the ampullae.

Again, treating the sensory epithelium as a “black box,” the firing process is modeled with an ad hoc firing rate gain function based on the known electrophysiology literature. The model consists of a firing rate function *r_i_(t)* generated in a given ampulla by the corresponding *V_signal_*. The index *i* refers to a specific ampulla–canal system, and *t* represents time.

Firing rate functions *r_i_(t)* are obtained by multiplying the indexed *V_signal_* values by a universal empirical gain function. To build this gain function, we note that the resting tonic rate in the absence of electric fields is known to be in the range of 30 to 40 Hz [11]. Data from the thornback ray suggest 100% change in rate for each 5 μV of apical voltage fluctuation [19], where negative drops are excitatory and positive fluctuations are inhibitory, with a somewhat sigmoidal overall shape.

**Figure 4:**
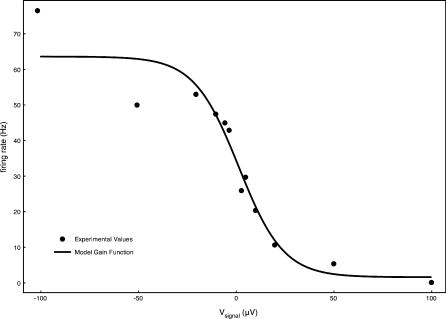
Firing Rate Gain Function Used for Computing Neural Activity in the Primary Afferent Fibers Associated with the Ampullae Available experimental data [11] was fitted to the sigmoid function 1.6 + 62 / (1 + 0.9 × exp(V_signal_/11.5)).

While those points provide anchors for the ampullary gain function, the shape was derived by digitizing the experimental points found in electrophysiology experiments with skates [11], to which we applied the standard Levenberg-Marquardt algorithm for nonlinear fitting. The resulting canal gain function can be seen in Figure 4.

In this treatment, we factor neither the natural relaxation of voltage signals within the ampullary system nor the natural relaxation of firing rate alteration in the primary afferents. These relaxations will be dominated by the rapidly developing electric potential changes over the short time scales of the skate–dipole interaction (the most dramatic activity in the results that follow include 1–2 s of closest approach). As noted for weakly electric fish, electric perception is limited to such short range that instantaneous data are presumably of fundamental importance [31].

In addition to raw firing rate data, and to further explore the potential fates of firing rates entering the DON, we use the concept of firing-rate population vector, [32] which was used successfully in the prey location analysis for the sand scorpion and the clawed frog [2,3].

We use a basic form of a population vector, where its heading (or yaw) in the horizontal plane of the skate is determined by a weighted average of the headings of the various canals. The weighting of each canal orientation is simply based on its electrosensor's firing rate. We thus consider a canal with heading *θ* to correspond to a unit vector (cos *θ*, sin *θ*) on the canal plane. We do this for both the right and left canal clusters. The population vector (p_x_, p_y_) is then given by the weighted sum of individual unit vectors:

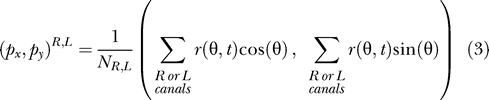



We take this vector to be a kind of compass heading that can bias the animal's orientation. In other words:

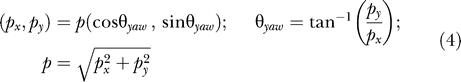



While we cannot confirm that the DON actually computes such a population vector, it shows, at the very least, what information is available to the skate from its peripheral electrosense.

## Results

**Figure 5:**
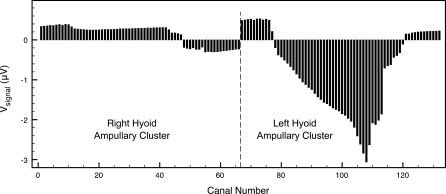
Relative Ampullary Electric Signal Snapshot for a Skate Approaching a Source that Is 30 cm to Its Left and 10 cm in Front of It Canal numbers correspond to those shown in Figure 1.

To illustrate the first step of the calculations, we present an example “snapshot” of signal voltages for the skate near a source. Figure 5 displays potential differences developing along the canals in Figure 2 for a skate approaching a source that is 30 cm to its left and 10 cm in front of it (as in Figure 3). Naturally, canals in the left hyoid ampullary cluster show the stronger signals.

**Figure 6:**
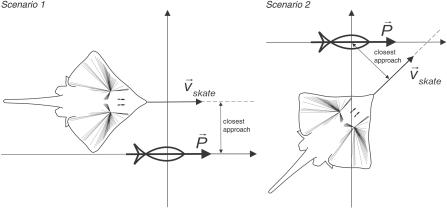
Two “Swim-By” Scenarios Used in the Simulations

We now present two example “swim-by” scenarios, with a skate swimming in a straight path past a nearby source (Figure 6). The responses presented here do not assess orientation behaviors; instead, they monitor the sensory information available to the skate as it moves past a bioelectric signal.

In the first scenario, a skate swims at 0.5 m/s past a source dipole with a closest approach distance of 0.15 m, in a direction parallel to the orientation of the source's dipole. In the second scenario, a skate swims again at 0.5 m/s, but with a direction no longer parallel to the source's dipole (45°). For comparative purposes, the closest approach distance is again set to 0.15 m.

**Figure 7:**
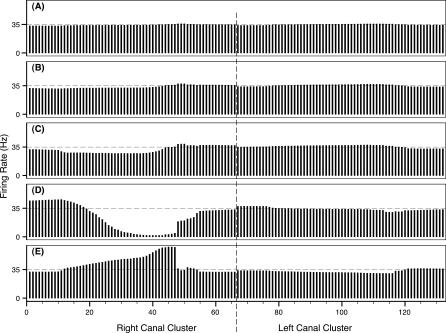
Firing Rate Snapshots, Representing the Instantaneous System Activity for Swim-By Scenario 1 Snapshots when skate–source distance was 0.50 m (A), 0.35 m (B), 0.25 m (C), the closest approach of 0.15 m (D), and 0.20 m after the closest approach (E). The abscissa refers to the canal numbers described in Figure 2. The ordinate refers to the firing rates associated with each ampulla. Dashed line indicates resting discharge rate.

**Figure 8:**
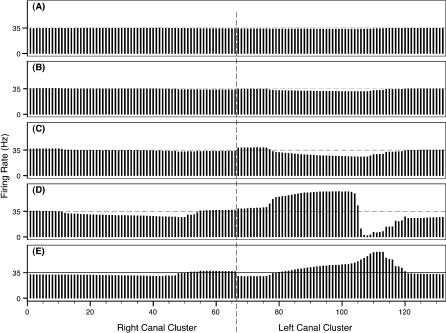
Firing Rate Snapshots, Representing the Instantaneous System Activity for Swim-By Scenario 2 Snapshots when skate–source distance was 0.50 m (A), 0.35 m (B), 0.25 m (C), the closest approach of 0.15 m (D), and 0.20 m after the closest approach (E). The abscissa refers to the canal numbers described in Figure 2. The ordinate refers to the firing rates associated with each ampulla. Dashed line indicates resting discharge rate.

As the skate moves in relation to the source in each of the two swim-by scenarios, the electric signal *V_signal_* associated with each electrosensory canal will change. Consequently, the associated firing rates also become functions of time (i.e., a function of environmental variables such as skate–source distance and relative orientation). Figures 7 and 8 present firing rate snapshots taken at different moments in each scenario.

As seen in Figure 6, canals in the right cluster for scenario 1 are closer to the source during the entire swim trajectory, and their associated firing rates show the largest variations, as depicted in Figure 7. Note that at skate–source distances greater than 0.30 m, each firing rate is essentially at the baseline value of around 34 spikes/s.

As the separation distance decreases, the canals' electric signals change in a nonuniform way. This leads to nonuniform firing rate profiles associated with each canal, as dictated by the firing rate gain function of Figure 4, with the maximum change from the baseline firing occurring around the closest skate–source approach.

Analogous to the previous case, canals in the left cluster for scenario 2 are closer to the source during the entire swim-by, and their associated firing rates show the largest variations, as depicted in Figure 8. We again note that at skate–source distances larger than 0.30 m, each firing rate is essentially at the baseline value.

**Figure 9:**
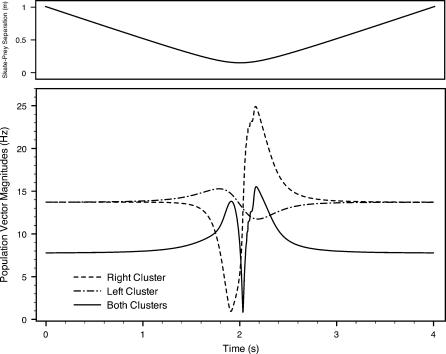
Population Vector Magnitudes and Skate–Source Separation Distance (Top Panel) for Scenario 1

**Figure 10:**
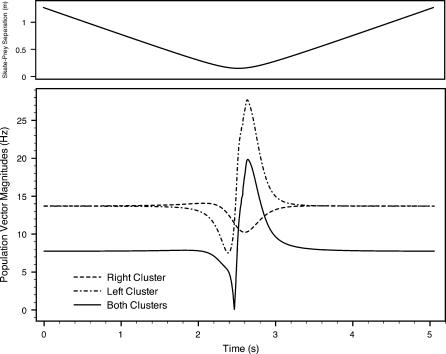
Population Vector Magnitudes and Skate–Source Separation Distance (Top Panel) for Scenario 2

To further explore how much neural information is available to the skate, we compute a firing rate population vector for each dorsal hyoid ampullary cluster (Equation 3). Figures 9 and 10 depict the resulting population vector magnitudes versus time for each scenario, charting the 4 to 5 s surrounding the point of closest approach. We depict the global or net population vector (i.e., summing for all hyoid canals) as well as the bilateral left and right clusters individually. Inputs from left and right are not combined in the neural architecture until they reach the skate's midbrain, so the left and right vectors may be relevant to the DON (e.g., processing common mode rejection). Also, note that the most significant variations occur within a total time of approximately 1 s.

**Figure 11:**
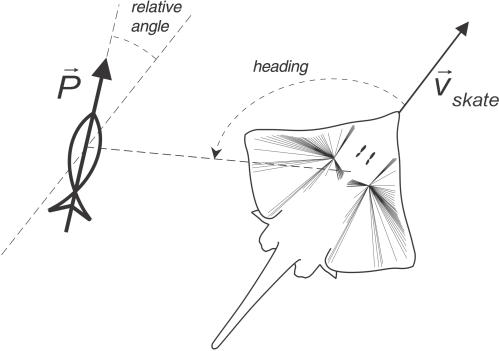
Angular Measures in the Skate–Source Geometry To prevent multivaluedness issues, we define all angles in the usual mathematical convention (i.e., 0°−360° range, counterclockwise with respect to the skate's longitudinal axis).

Like any vector, the population response includes angular (or heading) information, which we relate to the actual heading of the source (defined here as the angle between the skate's direction of motion and the vector pointing to the source position). Relevant angles are depicted in Figure 11. It is important to note that the dipole angle and the direction of the skate's motion remained fixed in each of the swim-by scenarios, while the heading of the source would naturally vary given the skate's motion.

**Figure 12:**
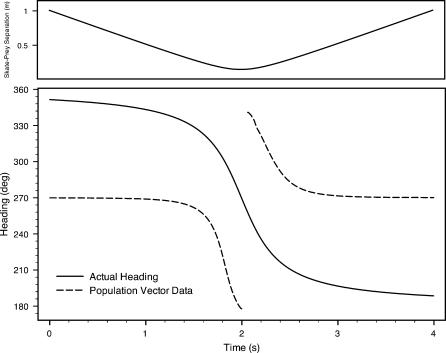
Net Population Vector Heading Data and Actual Source Heading versus Time for Scenario 1 The top graph depicts the separation distance over time. For this plot, a constant phase of 90° was added to the population vector data (see text).

**Figure 13:**
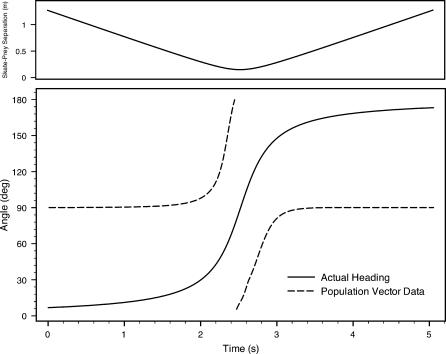
Net Population Vector Heading Data and Actual Source Heading versus Time for Scenario 2 The top graph depicts the separation distance over time. For this plot, a constant phase of 90° was subtracted from the population vector data (see text).

Figures 12 and 13 depict heading information from the global or net population vector. We plot the vector headings and the *actual* heading of the source dipole relative to the skate over time during each encounter. Of note are the abrupt discontinuities when the skate reaches the point of closest approach to the source, and the near perfect match (up to a constant phase) to the actual heading. In all of our simulations, population vector headings were defined with respect to the skate's longitudinal axis, following the usual counterclockwise mathematical convention. We note that such a human-centric system of angle definition need not bear any relation to the skate's neural processing of its electrical landscape. Hence, we believe constant offset phases (e.g., 90° alternately added to Figure 12 and subtracted from Figure 13), can be considered without decreasing the predictive nature of the modeling data. In other words, we do not assert that the net population vector should act like an orientation compass toward the dipole source. Rather, we point to relevant angular information encoded by the neuronal population's spiking activity.

Finally, we suggest experimental tests of the model and its assumptions. The following analysis can be tested directly by benchtop electrophysiology measurements in which investigators use dipoles in various positions to map receptive fields in anesthetized skates (e.g., [15]). We note that existing data do not track dipole *orientation*.

**Figure 14:**
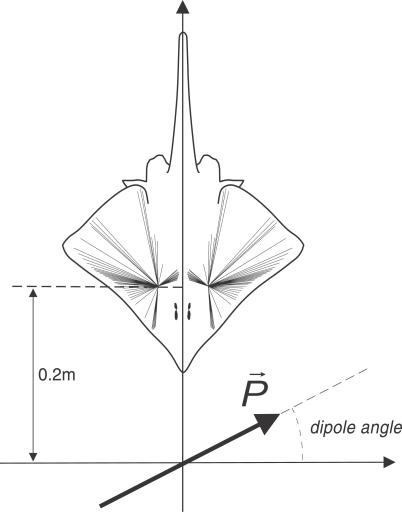
Geometric Arrangement for the Simulated Skate–Dipole Benchtop Experiment

In this simulation, a dipole is placed near a stationary skate, and the dipole is simply rotated in the horizontal plane of the skate without changing its position (Figure 14). This experiment has not been conducted, to the best of our knowledge. By monitoring the resulting firing rate activity and computed population vectors, we can predict “sweet spots” or regions in which the electrosensory system of the skate should show specific and varied reactions to relatively small changes in dipole orientation.

**Figure 15:**
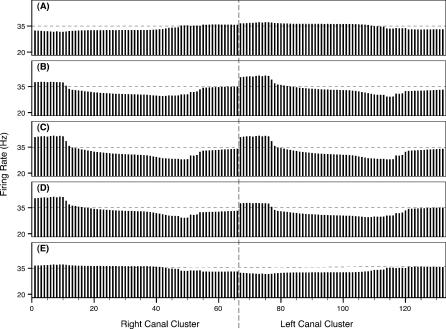
Firing Rate Snapshots Representing the Instantaneous System Activity for the Simulated Benchtop Experiment of Figure 14 The external electric dipole angle is 0° (A), 45° (B), 90° (C), 135° (D), and 180° (E). The abscissa refers to the canal numbers described in Figure 2. The ordinate refers to the DON firing rates associated with each one of these canals.

Given the sharp dependence of our modeling system on the interplay of the canal geometry with the dipole field, a significant variation in neural response results. The firing rate snapshots of Figure 15 show differences that arise in the ampullae simply by changing the relative angle between the dipole and the skate. Such sharp contrast would not follow from a model where pore potentials were communicated directly to the apical side of sensing cells [25–27]. Slight rotations do not change pore magnitudes of electric potential as appreciably as they change the canal potential differences considered in our model.

**Figure 16:**
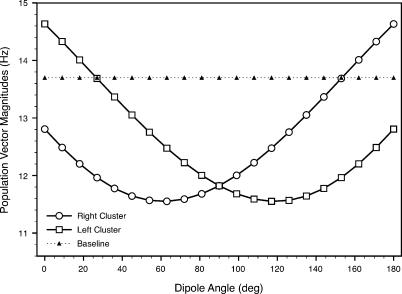
Population Vector Magnitude as a Function of the External Dipole Angle, as Defined in Figure 14 The “baseline” curve represents the magnitude of the population vector in the absence of an electric dipole field.

Another way to exhibit the spiking activity dependence on geometry is to plot the magnitude of the population vector for each canal cluster versus the external dipole angle (Figure 16). Here, as in Figures 9 and 10, the population vectors for both canal clusters have a magnitude of around 13.7 Hz when the source is far away or absent (i.e., *V*_signal_ for each canal is essentially zero). The maximum firing rate variation corresponds to an external dipole angle around 60° for the right cluster and 120° for the left cluster. The opposing nature of the geometric relationship between the skate and the dipole creates a predominance of positive electric potential signals, which have an inhibitory effect on firing rates (see Figure 4). A similar but excitatory result would be obtained if the external dipole were behind the skate, rather than in front of it.

## Discussion

**Figure 17:**
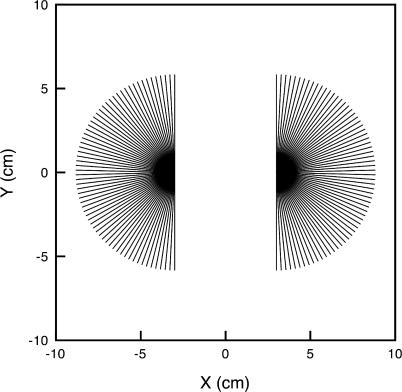
An Artificial Array of Canals for a Hypothetical Dorsal Hyoid Cluster

**Figure 18:**
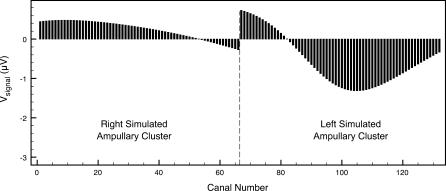
Relative Ampullary Electric Signal Snapshot for the Artificial Array Approaching a Source that is 30 cm to Its Left and 10 cm in Front of It

Though bilaterally symmetric, the canal arrays are asymmetric on either side as one moves from the anterior to posterior regions of the skate. This has a dramatic effect on the encoding of neural data (e.g., Figure 5), and we wish to emphasize two features in particular. First, the suppressed and fairly uniform signals exhibited by ampullae in the right cluster (farther from the dipole) are dramatically different from those of the left cluster (closer to the dipole). This type of uniformity in the contralateral signals would not emerge if the ampullae responded directly to pore potentials, and it does not emerge in data from a homogeneous array (see Figures 17 and 18 below). Second, there is an abrupt reversal of signal polarity for the short groups of canals on each side that project medial on the body (canals 55–66 and 121–132 in Figure 2). These subgroups effectively mimic the opposite cluster; such a feature could presumably be of great use to the skate in common mode suppression or signal amplification at higher levels of neurosensory processing.

For the firing rates computed, it is important to note that the variations revealed by our simulations are of significant magnitudes for even a simple neuronal system to respond accordingly. In a strict mathematical sense, this nonuniform firing rate profile encodes enough information for a precise location of the source, although empirical neurophysiological evidence is required to determine the processing of this information by the skate central nervous system.

The two main swim-by scenarios present fundamentally different cases. In the first, the source and the skate are aligned, and the source sits to the right of the skate's path; in the second, the source and the skate are not aligned, and the source sits to the left of the skate's path. While the skate clearly receives different information in these two scenarios, the global qualitative similarities of the firing rate snapshots for scenarios 1 and 2 overwhelm their differences.

First we note that firing from ampullae on the side opposite of the source are nearly unaffected, even when the skate passes within 15 cm of the source. This corresponds to the left cluster in Figure 7 and right cluster in Figure 8, and is consistent with the relative potential data shown for the right cluster in Figure 5. Presumably, such consistent lack of excitation or inhibition would be very beneficial to the skate's source location because it will provide a reference for contralateral excitation and inhibition by the source bioelectric field. Again, this is not the case for an artificial array of homogeneous canals.

Next, we note the dramatic changes for steps D and E for each trial. A sharp pattern of excitation and inhibition arises for the cluster nearest the source during the approach (Figures 7D and 8D). While some of the more anterior organs (e.g., canals 1–15 in Figure 7) show firing excitations, ampullae corresponding to the more posterior canals exhibit nearly complete inhibition (e.g., canals 35–45 in Figure 7).

As the skate passes and moves away from the source, consistent excitation emerges across the ipsilateral side. In particular, note that the organs experiencing inhibition on approach shift abruptly to excitation. In Figure 7D and 7E, for instance, organs 35–45 shift from near total inhibition to near 100% excitation over a travel distance of only a few centimeters by the skate. Not only would sharp changes provide for simple interpretations, the rate of change demonstrated in these simple trials fits the response characteristics of the ampullae. Given the skate's speed of 0.5 m/s, the organs in question experience this dramatic change over about 0.1 s, or at a frequency of 10 Hz. The time signature of this signal is within the ideal ampullary bandwidth of these organs [11,19]. And when considering the natural relaxation of firing rate alterations—afferent rates typically accommodate to a constant stimulus in less than 5 s [10]—the relatively quick changes shown in our trials again are within these neurophysiological constraints of the skate primary afferents. Both trials demonstrate asymmetry between approach and retreat situations, thus providing clear information concerning the anterior versus posterior source location.

We note with interest that the largest excitations were observed for sources in anterior locations to the body and after the skate passed the dipole source. This observation is consistent with the functional subunit hypothesis [17], and indicates that the dorsal hyoid clusters may best detect and encode information concerning anterior bioelectric sources. As skates are not just predators but are also potential prey, our results suggest that these clusters may be used to detect the approach of a large predator or conspecific. The dorsal location adds weight to this speculation.

In terms of electric source localization, the net population vector headings appear in Figures 12 and 13. Despite very different geometric scenarios in scenarios 1 and 2 (e.g., opposite relative source-to-skate position and orientation), the data relative to the actual source headings are virtually identical. The population vector headings follow the *change* of the actual source headings with precision, with two notable deviations. First, the population vector headings do not match the exact values of the source headings; second, an abrupt shift of direction occurs at the point of nearest approach. The fact that the precise angle values of the computed vectors do not match the apparent source headings is of very little concern, since we have imposed a human-centric system of angle definition (e.g., Figure 11). Moreover, a skate could presumably compensate for such regular offsets, much as visual processing inverts the actual image on the retina.

The abrupt shift at the point of closest approach (at the 2-s mark) coincides with the magnitude of the net vector falling briefly to zero. (See solid lines for net vector magnitudes in Figures 9 and 10.) We have confirmed that these shifts are absolutely independent of our choice of reference frame—the shifts are not an artifact of our angle definitions. As the skate moves from one side of the source dipole to the next, such a shift could be of significant biological benefit. The abrupt changes of vector magnitude and direction presumably give the skate a very clear signal as it comes very close to a prey, predator, or mate, and transitions from approach to moving away.

In summary, the skate electrosensory array can provide a wealth of information on the heading of a nearby bioelectric source by using a simple population vector scheme. The morphology is well suited to tracking the source location, including information concerning whether the skate is moving toward or retreating from a source. In addition, population vector magnitudes vary sharply at the position of closest approach. This presumably supplies clear neural information that the source is within critical, minimum distance associated with the given swimming trajectory. Moreover, the changes in firing activity happen over a time scale that is ideally suited to the frequency response of the electrosensors.

It is not unreasonable to suggest that the overall shape of R. laevis evolved at least in part for the tuning of electrosensory tasks, especially as the dorsal hyoid canal pores extend to the periphery of the skate's wings (Figure 2). The location of the hyoid pores follows this pattern for the canal systems of most skates [24].

Does the skate morphology confer an obvious advantage over, for instance, a simple homogeneous array of canals? We ran the same simulations for an artificial set of dorsal hyoid electrosensors with associated canals of uniform 10-cm lengths and equal angular spacing covering 360° of horizontal arc.

Figure 17 depicts a homogeneous array of 132 canals, with 66 per cluster, as in the skate (compare with Figure 2). The two clusters have the same lateral spacing as those in the skate. The canal lengths of the artificial array are set to 10 cm, the average length of those for the skate's dorsal hyoid cluster; the angular distribution is similarly uniform, with canals placed every 2.78°, as opposed to the skate's actual canals, which have some densely spaced canals in terms of orientation, while some others are spaced more broadly.

We compute a snapshot of *V_signal_*, according to Equation 2, for each of the canals, exactly as we did for the skate array. The results are shown in Figure 18 (compare with Figure 5). The significant differences for the skate versus the homogeneous, artificial array are as follows. The skate signals are, naturally, of much greater range, reflecting the range of canal lengths—the signals of the skate's system are three times that of what a homogeneous array would offer. Even if one adjusts the canal lengths of this artificial array to the maximum skate canal length, however, a more fundamental difference remains. The canal-to-canal signal differences vary in a moderate fashion for the artificial array, while those in the skate vary dramatically, even for some nearly adjacent canals. Figure 5 exhibits signals that change by approximately 50% over as few as three canal spacings, while Figure 18 shows that a similarly dramatic difference can only be obtained over 15–20 canal spacings.

Further computations of firing profiles and population vector magnitudes exhibit the same fundamental differences shown in Figure 18. The size of signals are typically smaller for the artificial canal array, and in all cases the signals change more gradually, as shown both over the array of canals, and also over time, in the case of the population vector magnitudes.

These trials confirm that a homogeneous array can also track the source dipole, but at least two distinct advantages for the skate's varied canal morphology emerge. First, the “snapshot” canal voltages (see Figure 5 and Figure 18) are much less dramatic for the artificial array; the contralateral cluster does not exhibit a homogenous set of voltages, and the variation of signals within the near-side cluster exhibits a much less pronounced change. In essence, the homogeneous, artificial array would not provide the same sharp contrast of signals between organs. Second, though firing rates and population vectors change for the artificial array, they change more slowly over time. As noted previously, referencing Figure 7, a subset of canals can change their firing rate from near 0 to 50 Hz over about 0.1 s as the skate moves past the dipole. Similarly, the skate's morphology provides population vector magnitudes that nearly double in less than 0.25 s, while a similar change in the artificial array takes a second or more. This is crucial when one considers that the electrosensory system of the skate is finely tuned to changes in a narrow bandwidth peaked between 2 and 10 Hz [10–12,19]. Hence, an abrupt change evolving in a fraction of a second would be much more useful than a change that takes a second or more.

To correctly model the function of the electrosensory periphery, it is critical to understand the excitation of canal arrays by bioelectric fields. We have proposed a simple, specific test for the modeling scheme advocated here. We identify an area of specific opposite predictions from our model and that of cable-like, equipotential canal function. As the dipole of Figure 14 turns between 0°–90°, the anterior pore potentials (e.g., for canals 0–50 in the left cluster) become increasingly positive, via the dot product of Equation 1. Hence, a cable-like canal function, conveying the pore potential to the ampullae, would predict *inhibited* firing rates for increasing apical potentials. However, our model predicts the opposite, as can be seen in Figure 15A–15C. The turn between 0° and 90° yields negative *V_signal_* values and *excited* firing rates. Furthermore, according to the data shown in Figures 15 and 16, a quick dipole rotation from just 0° to 45° would appreciably alter not only the firing rates associated with the anterior canals but also the population vector magnitudes. These changes could be exhibited in primary afferent rates, ventilatory activity, heart rate, or even DON activity. A traditional theory by which canals simply convey pore voltage signals to the ampullae would not be as sensitive to the canal's overall alignment with the dipole field vectors.

We have modeled the neural responses of only a small subset of the approximately 1,400 ampullary canals in the barndoor skate. While we chose the approximately 132 hyoid canals that project to the dorsal surface of the skate's body for their apparent sensitivity and to present clear and finite data, future efforts incorporating all clusters are required to understand central processing and integration of receptor population data. Even more dramatic and discontinuous rostral and caudal projections are found in the dorsal canals of the skate superficial ophthalmic cluster [14]. In addition, there are approximately 700 total hyoid canals that project to the ventral surface. These canals are more continuous in distribution, and have prominent contralateral projections that cover a wider angular field. Even more dramatic differences in canal projection vectors exist among the dorsal and ventral buccal clusters. Application of the electrodynamic model that integrates responses of these ampullary groups would provide a more comprehensive model of electrosensory processing of environmental fields in feeding, social behavior, and navigation. In addition, it would permit development of models for testing the functional subunit hypothesis [14], in which information from subgroups of canals in different clusters that have similar directional projections may be integrated to maximize direction computations.

Future comparative work that assesses electrosensory-processing mechanisms across taxa will also provide important clues on the evolution and diversity of ampullary arrays seen in elasmobranch fishes.

## Abbreviations

DON: dorsal octavolateralis nucleus
